# The determination of the optimal threshold on measurement of thyroid volume using quantitative SPECT/CT for Graves' hyperthyroidism

**DOI:** 10.1186/s40658-023-00608-w

**Published:** 2024-01-05

**Authors:** Chengpeng Gong, Yajing Zhang, Fei Feng, Mengmeng Hu, Kun Li, Rundong Pi, Hua Shu, Rongmei Tang, Xiaoli Wang, Shilin Tan, Fan Hu, Jia Hu

**Affiliations:** 1 Department of Nuclear Medicine, Union Hospital, Tongji Medical College, Huazhong University of Science and Technology, Wuhan, 430022 China; 2 Hubei Province Key Laboratory of Molecular Imaging, Wuhan, 430022 China

**Keywords:** SPECT/CT, Phantom, Graves’ hyperthyroidism, Thyroid volume, Na^99m^TcO4

## Abstract

**Purpose:**

To investigate the optimal threshold for measuring thyroid volume in patients with Grave's hyperthyroidism (GH) by SPECT/CT.

**Materials and methods:**

A 53 mL butterfly-shaped hollow container made of two 45-degree transparent elbows was put into a NEMA IEC phantom tank. The butterfly-shaped container and the tank were then filled with Na^99m^TcO4 of different radioactive concentrations, respectively, which could simulate thyroid gland with GH by different target-to-background ratios (T/B) (200:1, 600:1, 1000:1). The different T/B of planar imaging and SPECT/CT were acquired by a Discovery NM/CT 670 Pro SPECT/CT. With Thyroid software (Version 4.0) of GE-Xeleris workstation, the region of the thyroid gland in planar imaging was delineated. The thyroid area and average long diameter of both lobes were substituted into the Allen formula to calculate the thyroid volume. The calculation error was compared with the actual volume. Q-Metrix software was used to perform CT-based attenuation correction, scatter correction, resolution recovery. Ordered-subsets expectation maximization was used to reconstruct SPECT data. 20%, 25%, 30%, 40%, 50%, 60% thresholds were selected to automatically delineate the volume of interest and compared with the real volume, which determinated the optimal threshold. We measured the thyroid volume of 40 GH patients using the threshold and compared the volumes obtained by planar imaging and ultrasound three-dimensional. The differences of the volumes with different T/B and thresholds were compared by the ANOVA and least significant difference *t* test. The volumes delineated by SPECT/CT were evaluated using ANOVA, least significant difference *t* test, correlation analysis and, linear regression and Bland–Altman concordance test plot. The differences and consistency of thyroid volume were compared among the above three methods.

**Results:**

There was no significant difference in the results between different T/B models (*P* > 0.05). The thyroid volume calculated by the planar imaging formula method was higher than the real volume, with an average overestimation of 22.81%. The volumes delineated by SPECT/CT threshold automatically decreased while the threshold increased. There were significant differences between groups with different thresholds (*P* < 0.001). With an average error of 3.73%, the thyroid volume analyzed by the threshold of 25% was close to the results of ultrasound measurement (*P* > 0.05). Thyroid volume measured by planar imaging method was significantly higher than ultrasound and SPECT/CT threshold automatic delineation method (*P* < 0.05). The agreement between the SPECT/CT 25% threshold and ultrasound (*r* = 0.956, *b* = 0.961) was better than that between the planar imaging and ultrasound (*r* = 0.590, *b* = 0.574). The Bland–Altman plot also showed that the thyroid volume measured by the 25% threshold automatic delineation method was in good agreement with the ultrasound measurement.

**Conclusions:**

The T/B has no effect on the measurement of thyroid volume in GH patients; planar imaging method can significantly overestimate thyroid volume in GH patients, and 25% threshold automatic delineation method can obtain more accurate thyroid volume in GH patients.

## Introduction

^131^I has been used to treat hyperthyroidism since 1941 [1], it is one of the first-line treatment for Graves’ hyperthyroidism (GH) [2]. The dose of ^131^I is usually determined by the calculated dose method or the fixed dose method [3]. Compared with the fixed dose, the calculated method can reduce the dose of ^131^I, consequently reduce radiation damage of patients, conforming the concept of precise and individualized treatment. The dose of ^131^I is proportional to the volume of the thyroid gland [4–6] in the dosimetry method; therefore, the accurate determination of the thyroid volume is very important to determine the therapeutic dose of ^131^I. At present, the methods for thyroid volume mainly include: palpation, radionuclide imaging [7], ultrasound, CT, MRI [8] and PET/CT [9, 10]. Radionuclide imaging is a widely used method to obtain quantitative functional and volumetric information. Due to the lack of depth, the accuracy of planar imaging has been questioned [5]. The measurement of thyroid volume by planar imaging is greatly affected by the operator's experience. Even if the same operator repeats the operation, the repeatability is still suboptimal. Therefore, finding a more accurate and stable radionuclide imaging method has become a research hotspot.

With the continuous improvement of photon correction technology and iterative reconstruction algorithm of integrated SPECT/CT, quantitative technology has been achieved in SPECT/CT [11]. Previous studies [12–16] reported application of quantitative SPECT/CT in the diagnosis and treatment of thyroid diseases. Some researcher teams have stated that the thyroid volume of interest (VOI) can be delineated by automatically extracting the pixel points that are higher than the maximum uptake value and the threshold value and obtained the standardized uptake value (SUV) and volume of the thyroid in one step [13]. Although the feasibility of the method and the clinical significance of quantitative parameters were described, the selection of the threshold was not discussed in this study, which is an particularly important parameter [13]. In this research, a butterfly-shaped phantom is constructed to simulate the physiological characteristics of the GH. The optimal threshold for GH thyroid volume was determined using SPECT/CT quantitative method according to different T/B. Furthermore, the optimal threshold will be validated further in clinical data.

## Materials and methods

### Equipment and preparation of the simulated thyroid model

Discovery NM/CT 670 Pro of GE Company was used, equipped with 16-slice spiral CT, low-energy and high-resolution collimator. For radioisotope ^99m^Tc, tomographic spatial resolution (without RR) of 10.59 mm and a system sensitivity (^99m^Tc) of 73.4 cps/MBq, which were measured according to the NEMA guidelines. The software for quantitative analysis was Q.Metrix of GE-Xeleirs 4.0 workstation (GE Healthcare. USA). Na^99m^TcO4 was supplied by Wuhan Atomic Hi-Tech Co., Ltd. CRC-25R type radioactivity meter (CAPINTEC, USA) was applied to measure the radioactivity.

**Fig. 1:**
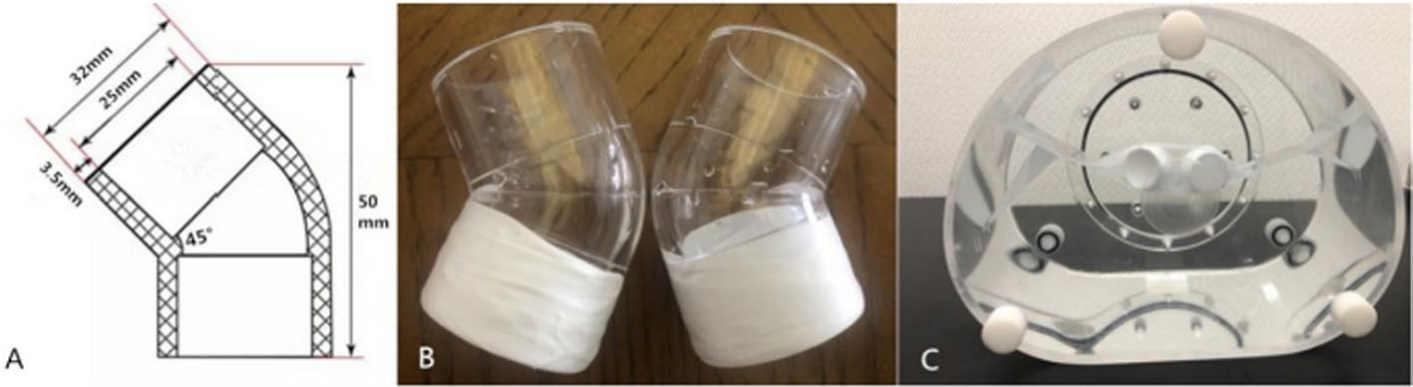
Thyroid model. **A** Single elbow size, **B** bilateral lobe model, **C** specific T/B thyroid model

The thyroid model was made by bonding two 45-degree transparent elbows (Zaozhuang Yicai Trading Co., Ltd.), and the two ends of the elbows were sealed with waterproof tapes after filling with liquid. The inner diameter of a single elbow was 25 mm, the thickness of the wall was 3.5 mm, with 32 mm of the outer diameter and 50 mm of the height. The volume of the unilateral elbow in pure water was 26.5 mL, so the volume of the bilateral elbows was 53 mL, as shown in Fig. 1A, B. It was placed horizontally inside the NEMA IEC model (Data Spectrum, USA) from which the component and the ball were taken out. The volume of the model tank was 10,122 mL measured with pure water. Firstly, we kept the phantom dry. Then, we dissolved 0.6 mL of freshly rinsed 148 MBq Na^99m^TcO4 in 103 mL of water. 53 mL was taken out and filled into the bilateral lobes of the thyroid gland after well-mixed, with the radioactive concentration 142,857 Bq/mL. The remaining 50.6 mL of the solution was poured into the tank, and then, the tank was filled with water. The radioactive concentration of the tank was 714 Bq/mL. Regarding thyroid model as the target area and the tank as the background, a thyroid model with a tomographic T/B 200 was formed. The activity in the thyroid phantom was kept constant, and the background was diluted, the tomographic T/B 600 and 1000 models were prepared, as shown in Fig. 1C.

### SPECT and SPECT/CT acquisition and image processing

The prepared thyroid model was placed on the examination bed, the bed height adjusted to coincide the cross light with the center of the model. Low-energy high-resolution (LEHR) collimator, energy peak (140 keV, window width 10%), matrix 256 × 256 and zoom 4.0 were selected for planar imaging. Different counts (300 Kcts/frame, 500 Kcts/frame and 800 Kcts/frame) were collected for each frame. The planar imaging data were analyzed by thyroid post-processing software (GE-Xeleirs workstation with a Version of 4.0). The GE ‘PHAGE PHASE’ blue boundary was used for manual delineation as the target and background, subsequently obtained the frontal projection area of the thyroid *S* (cm^2^), the average height of the thyroid in both lobes *L* (cm), and the planar imaging T/B (thyroid radioactivity count/background radioactivity count). According to the formula (1) [17], formula (2), we calculated the thyroid volume and compared it with the real volume (53 cm^3^).

1 $$V\left( {{\text{cm}}^{3} } \right) = S*L*K\left( {{\text{the}}\;{\text{use}}\;{\text{of}}\;{\text{a}}\;K\;{\text{constant}}\;{\text{of}}\;0.32} \right)$$

2 $${\text{Volume}}\;{\text{error}} = \left| {{\text{measured}}\;{\text{volume}} - {\text{real}}\;{\text{volume}}} \right|/{\text{real}}\;{\text{volume}}$$

SPECT/CT: main energy peak (140 keV, window width 10%), scatter window (120 keV, window width 5%), matrix 128 × 128, zoom 1.0, body contour, 6°/frame of 360° acquisition, three groups (12 s/frame, 15 s/frame and 18 s/frame, 60 frames in total) were collected for each image; 120 kV tube voltage, automatic exposure control, 1.25 mm slice thickness were selected for low-dose CT acquisition.

Additionally, SPECT images were reconstructed by using an iterative ordered subset expectation maximization (OSEM) algorithm (8 iterations and 10 subsets) with CT-based attenuation correction (CTAC), scatter correction (SC), and resolution recovery (RR), no post-processing filter. 20%, 25%, 30%, 40%, 50%, and 60% of the maximum uptake value of the target area were used as the thresholds to automatically delineate the VOI of the bilateral thyroid lobes in the Q.Metrix of GE-Xeleirs 4.0 workstation (GE Healthcare. USA), and the thyroid volume from each threshold was analyzed and compared with the true volume.

### Patient information

**Fig. 2:**
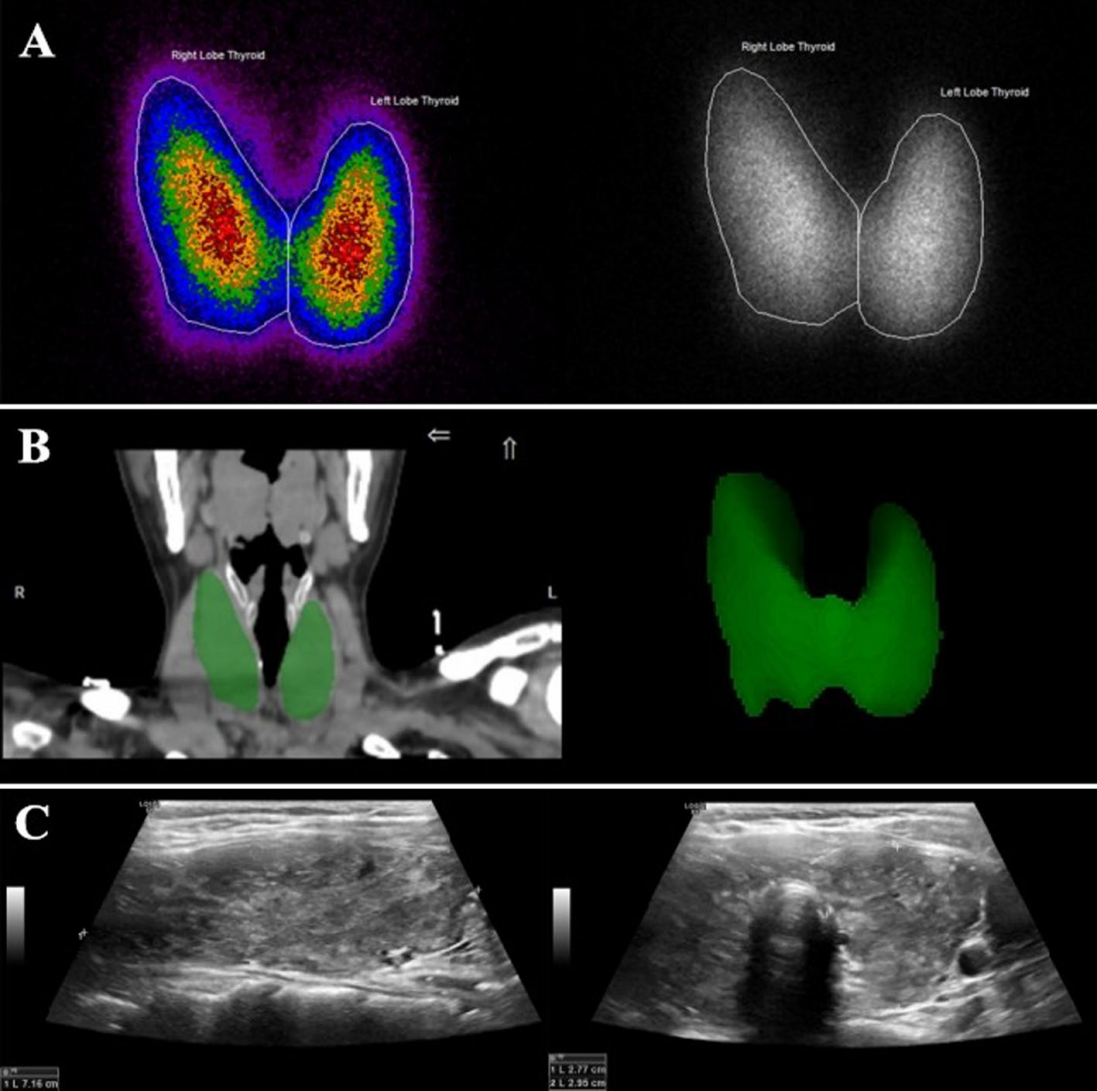
Schematic diagram of thyroid volume by three methods. **A** Planar imaging measurement method. **B** SPECT/CT threshold automatic delineation measurement method. **C** Ultrasound measurement of unilateral lobe length, width and thickness

We retrospectively analyzed 40 patients with clinically diagnosed GH who underwent thyroid radionuclide (Na^99m^TcO4) planar imaging and tomographic imaging in our department from March 2021 to September 2021 who underwent neck ultrasound within one week in our hospital. There were 10 males and 30 females, aged (32 ± 13) years old. All patients stop antithyroid drugs for one week before scanning and did not receive iodine contrast agent and other factors that affect thyroid function within one month, with a low-iodine diet 1–2 weeks before the examination. The patient was placed in the supine position 30 min after intravenous injection of 185 MBq Na^99m^TcO4, and the thyroid planar imaging and SPECT/CT tomographic fusion imaging were performed. Planar imaging acquisition 500 Kcts, tomographic imaging 15 s/frame, other acquisition parameters and volume measurement methods were the same as the model. Thyroid volume measurement was estimated by 3D ultrasonography using a linear 7.5-MHz probe. During the ultrasound examination, subjects lay in a supine position with the neck hyperextended and the shoulders was supported by a pillow. The length (L1, L2), width (W1, W2) and thickness (T1, T2) of left and right thyroid lobes were measured, respectively. Thyroid volume by ultrasound was calculated using the formula (3) [18]. Using the optimal threshold determined by the model, the patient's thyroid volume was automatically delineated on SPECT/CT scan and compared with the results measured by ultrasound and planar imaging methods. Three methods of thyroid volume measurement were shown in Fig. 2.

3 $$V\left( {{\text{cm}}^{3} } \right) = \pi /6 \cdot L1 \cdot W1 \cdot T1 + \pi /6 \cdot L2 \cdot W2 \cdot T2$$

### Statistical processing

SPSS 20.0 software was used for statistical analysis, and data conforming to the normal distribution was expressed as mean ± standard deviation. ANOVA and t test were used to compare the differences in volume measurement of thyroid simulation model among different T/B and different thresholds. ANOVA, *t* test and Bland–Altman concordance test were used to evaluate the differences and consistency of SPECT/CT threshold automatic delineation, ultrasonography and planar imaging in measuring thyroid volume in patients with hyperthyroidism. *P* < 0.05 was considered to be statistically significant.

## Results

### Differences in volume measurement with three target-to-background ratio models by planar imaging formula method

To calculate the volume of the thyroid gland by using the planar imaging formula method, three groups were prepared according to their T/B. All three groups were higher than the real volume, which were 67.50 ± 0.26 mL, 62.98 ± 2.56 mL, 64.79 ± 2.79 mL, with an average error of 22.81%, showing no significant difference among the three groups (all *P* > 0.05), and the planar imaging results of different T/B models were shown in Table 1.

**Table 1:** Planar imaging results of different T/B models

Group	Planar imaging T/B		Bilateral lobe projected area S(cm^2^)	Average length L(cm)	Measured volume V(mL)	Volume error (%)
1		6	34.18 ± 0.31	6.07 ± 0.05	67.50 ± 0.26	27.37 ± 0.48
2		16	32.54 ± 0.99	6.03 ± 0.04	62.98 ± 2.56	18.83 ± 4.84
3		24	33.13 ± 1.22	6.07 ± 0.03	64.79 ± 2.79	22.24 ± 5.26
	*F*		2.446	0.731	3.243	3.243
	*P*		0.167	0.520	0.111	0.111

### Differences in automatic delineation of the thyroid volumes with different thresholds and different T/B by quantitative SPECT/CT

There were no statistically significant differences in the volumes among three T/B groups with the same threshold (*P* > 0.05). As the threshold increased, the delineated volume decreased, showing significant differences between the groups with different thresholds (*P* < 0.001). 25% threshold was the closest to the real volume, with an average error of 3.73%. The results of automatic delineation of thyroid volume with different thresholds were shown in Table 2. Table 3 showed the error of automatic delineation of thyroid volume with different thresholds.

**Table 2:** The ANOVA and t test analysis of automatic delineation of thyroid volume with different thresholds

Group	Tomographic T/B		Automatic delineation of measured thyroid volume with different thresholds (mL)					*F*	*P*
			20%	25%	30%	40%	50%		
1		200	62.43 ± 1.10	54.97 ± 1.25	48.77 ± 1.16	39.27 ± 1.85	31.10 ± 1.99	200.94	0.000
2		600	62.33 ± 1.01	55.23 ± 0.87	49.90 ± 0.56	41.13 ± 0.61	33.20 ± 0.30	771.85	0.000
3		1000	61.67 ± 0.21	54.73 ± 1.10	49.30 ± 1.47	40.03 ± 1.95	31.87 ± 1.69	206.93	0.000
	*F*		0.686	0.16	0.757	1.043	1.468		
	*P*		0.539	0.856	0.509	0.409	0.303		

OSEM (8 iterations, 10 subsets), CTAC, SC, RR

**Table 3:** The ANOVA analysis of automatic delineation of thyroid volume errors with different thresholds

Group	Tomographic T/B		Automatic delineation of thyroid volume error with different thresholds (%)					*F*	*P*
			20%	25%	30%	40%	50%		
1		200	17.80 ± 2.07	3.71 ± 2.36	7.99 ± 2.19	25.91 ± 3.49	41.32 ± 3.76	82.51	0.000
2		600	17.61 ± 1.91	4.21 ± 1.65	5.85 ± 1.05	22.39 ± 1.16	37.36 ± 0.57	300.55	0.000
3		1000	16.35 ± 0.39	3.27 ± 2.07	6.98 ± 2.78	24.46 ± 3.68	39.88 ± 3.19	89.97	0.000
	F		0.686	0.16	0.757	1.043	1.468		
	F		0.539	0.856	0.509	0.409	0.303		

OSEM (8 iterations, 10 subsets), CTAC, SC, RR

### Difference and consistency of thyroid volume in GH patients measured by 25% threshold, planar imaging formula method and ultrasound method

The results of the thyroid volume of 40 patients with GH by three different methods demonstrated that automatic delineation method with 25% threshold was as accurate as the ultrasound results, showing no statistically significant differences (*P* > 0.05). Thyroid volume measured by planar imaging method was significantly higher than the other two methods (both *P* < 0.05). Compared with ultrasound, planar imaging showed an average overestimation of 25% [(39.64–31.68)/31.68]. The clinical data of 40 patients with GH and the thyroid volumes by three methods were shown in Table 4.

**Table 4:** Results of three different methods for measuring thyroid volume in patients with GH (*n* = 40)

	Ultrasound	Planar imaging	SPECT/CT 25% threshold automatic delineation
Volume (mL)	31.68 ± 15.04	39.64 ± 15.26	32.73 ± 15.12^a^
*F*		3.267	
*P*		< 0.05	

a: compared with ultrasound measurement, *P* = 0.810; compared with planar imaging, *P* < 0.05; Threshold automatic delineation method of SPECT/CT: CTASCRR, OSEM8i10s, 25% threshold

The correlation between SPECT/CT 25% threshold and ultrasound was well as shown in Table 5. The correlation between the SPECT/CT 25% threshold and ultrasound (*r* = 0.956, *b* = 0.961) was better than that between the planar imaging and ultrasound (*r* = 0.590, *b* = 0.574).

**Table 5:** Correlation analysis between planar imaging, SPECT/CT 25% threshold and ultrasound for estimation of the thyroid size in patients with GH

	A	B
Correlation coefficient (r)	0.956	0.590
Regression coefficient (b)	0.961	0.574

A. Correlation between SPECT/CT 25% threshold and ultrasound for estimation of the thyroid volumes in patients with GH. B. Correlation between planar imaging and ultrasound for estimation of the thyroid volumes in patients with GH

**Fig. 3:**
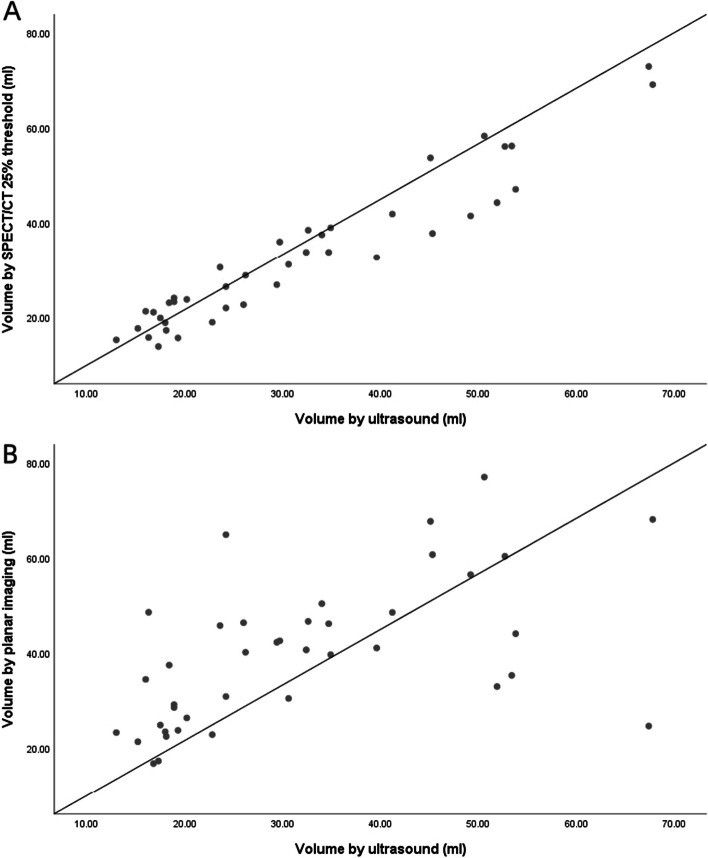
Correlation between the thyroid volumes estimated by planar imaging, SPECT/CT 25% threshold and ultrasound. **A** SPECT/CT 25% threshold and ultrasound. **B** Planar imaging and ultrasound

**Fig. 4:**
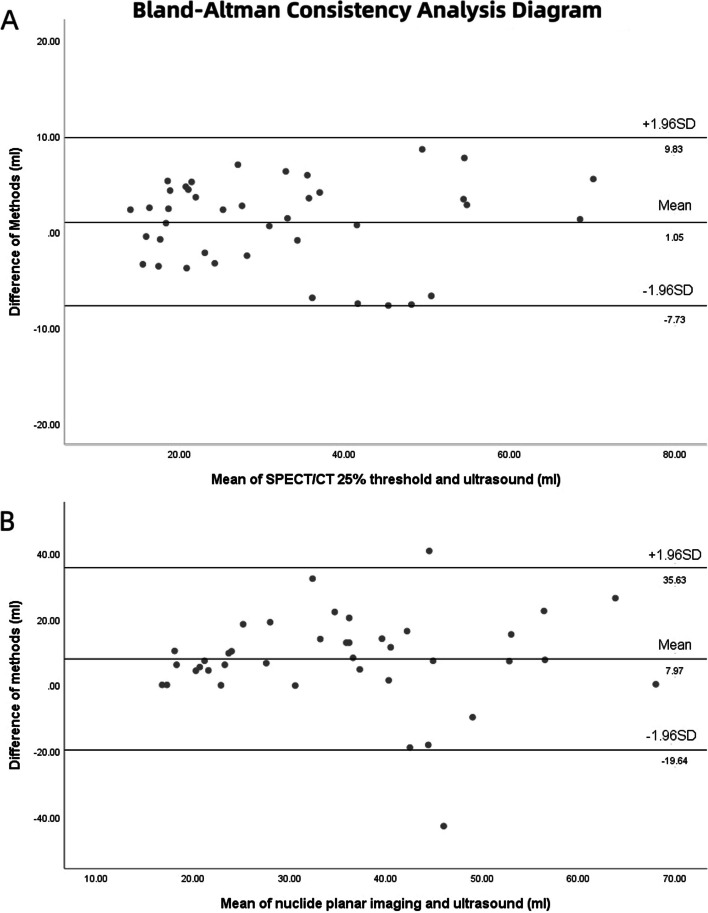
Bland–Altman Consistency Analysis Diagram. **A** Bland–Altman diagram of thyroid volume measured by SPECT/CT 25% threshold and ultrasound. **B** Bland–Altman diagram of thyroid volume measured by planar imaging method and ultrasound

The correlation of volume measured by SPECT/CT 25% threshold and ultrasound was well, which was shown in Fig. 3. The Bland–Altman diagram shows that the thyroid volume measured by the 25% threshold automatic delineation method was in good agreement with the ultrasound measurement method. Bland–Altman consistency analysis was shown in Fig. 4.

## Discussion

Among the common modalities for measuring thyroid volume in clinic, ultrasound, CT and MRI are three-dimensional imaging methods with difficulties in accurately reflecting the functional status of the thyroid glands. Although ^124^I-NaI PET/CT is an ideal imaging modality, the cost is too high [9, 10]. Planar imaging of thyroid radionuclide is simple and economical, which can simultaneously provide thyroid function status and thyroid volume. Therefore, it is a widely used thyroid mass assessment method in clinic. However, there are some factors affecting its accuracy in the measurement with planar imaging, such as thyroid size, geometry, gland thickness, and ratio of radioactive uptake between the gland and the surrounding background [7]. The SPECT/CT quantitative method can theoretically effectively compensate for these influencing factors. Nevertheless, the published studies are few, especially there is even no relevant literature about the threshold selection of relevant parameters. In this study, an self-made double-leaf butterfly-shaped container with a volume of 53 mL was placed in a NEMA IEC phantom tank to simulate the thyroid gland of patients with GH. The volume was compared with the real volume, and finally, the conclusions obtained were further verified in the clinical data. The methodology and conclusions were reasonable and reliable.

The model volume which was measured by the planar imaging formula method and the SPECT/CT automatic delineation threshold method was compared with the true value. Isselt et al [5] recommended to select a threshold value of 30% for 10–40 ml models to outline ROI, and we used 30% for 53 ml butterfly models. Our threshold delineation of the image had rougher boundaries and smaller measured volume, especially with inhomogeneous imaging agent, irregular thyroid gland or smaller T/B. It might be the special shape of our model, which also exceeded 40 ml in size, that caused the difference in results. Therefore, we chose the blue boundary of “PHAGE PHASE” as the standard manual delineation for delineating ROI (which was similar to the 20% threshold automatic delineation result, but the manual delineation boundary was smoother. We believed that manual delineation was more suitable for irregular shapes and thyroid glands with low T/B). The results showed that the planar imaging formula method significantly overestimated the thyroid volume, with an average overestimation of 22.81%, and the average error of SPECT/CT automatic delineation threshold to measure the thyroid volume was only 3.73%. The clinical data of GH patients also showed that the thyroid volume measured by the planar imaging formula was significantly higher than the thyroid volume measured by ultrasound and three-dimensional measurement of SPECT/CT. We have summarized at least 4 reasons: Firstly, the measurement of thyroid volume by ultrasound or radionuclide plane imaging is an empirical estimation formula based on regular mold (cylinder, ellipsoid). When the thyroid morphology of nodular goiter and multiple thyroid adenoma changes, the volume estimation error using the above method may increase. Secondly, regarding of the formula (1) for calculating the volume of planar imaging, *K* was a variable constant between 0.23 and 0.32 on the basis of specific and different instrument conditions. Allen and Goodwin [19] used a *K* factor of 0.128 when the frontal area of the gland was expressed in 0.25 inch squares and 0.323 when the area was expressed in square centimeters. We expressed the area in square centimeters, the *K* factor is fixed by 0.32 that may cause some degree of overestimation. Thirdly, there is still the influence from glandular isthmus. The larger area of the isthmus is, correspondingly the measuring volume is higher. But the isthmus is generally thinner than lobes, which may lead to overestimated results. Lastly, planar imaging was a two-dimensional imaging without depth information. Adipose tissue or tissues around the thyroid was inevitably included in the delineation of the target area, resulting in an overestimation of the frontal projection area of the 2 lobes and an overestimation of the thyroid volume.

GH is often manifested as an increased ratio of Na^99m^TcO4 uptake by the thyroid to the surrounding background. In this study, T/B was formulated at different of 200, 600, and 1000, and the corresponding T/B measured on planar imaging was 6, 16, and 24. There were no significant differences in the bilateral lobe area, average length and volume of the thyroid gland calculated by the planar imaging method among the three different T/B models. There was no significant difference in the thyroid volume measured by the SPECT/CT threshold automatic delineation method. It showed that there was no relation between the measurement of thyroid volume and T/B. In other words, the influence of T/B in the measurement of thyroid volume in patients with GH by radionuclide imaging was not significant.

Attenuation correction of SPECT data by low-dose CT was the basis of accurate quantification. However, compared with the increased radiation dose caused by overestimation of thyroid volume (increased dose caused from 131I), the increased radiation dose (less than 1 mSv) from low-dose CT is almost negligible. Quantitative SPECT/CT not only extraction of thyroid three-dimensional information and volume measurement but also quantification the thyroid function status, which is of great significance for the diagnosis and treatment of hyperthyroidism [13]. With this advanced processing method, the calculation of the target volume is simple and quick to achieve. Furthermore, since it is based on the threshold-based automatic segmentation, the segmentation results are only related to the distribution of radionuclides but not affected by thyroid morphology and operator. When the thyroid morphology of nodular goiter and multiple thyroid adenoma changes does not affect the volume measurement, but an underestimation of the volume may occur in the case of less hyperfunctioning nodules. It is particularly important that appropriate threshold selection was the premise of accurate measurement. Due to the existence of partial volume effect, the optimal threshold for target areas volume measurement of different sizes varies greatly. Pacilio et al. [20] obtained a Jaszczak model with a T/B of 14.4 by ^99m^Tc perfusion showed that the optimal threshold decreased with the increase in the target volume studied. When the target volume was 19 mL, the optimal threshold was about 40%, and when the target volume was 0.5 mL, the optimal threshold was even over 90%. Another study [17] proposed that the optimal threshold for measuring thyroid volume by SPECT was 30% or 35%. In this study, different thresholds were used to measure the volume of the thyroid model, and the results showed that as the threshold increased, the measured volume decreased. In our study, the volume of the thyroid model measured by the automatic delineation of the 25% threshold is the closest to the real volume. At the same time, clinical data also show that the thyroid volume measured by the automatic delineation of the 25% threshold has a high consistency with the thyroid volume measured by ultrasound. It is consistent with the above study that the threshold changes with volume, but the optimal threshold is slightly different from them [17, 20], mainly because we selected a target volume that was more capable of simulation of the thyroid volume in GH patients. The target area was thyroid shaped, the target volume was more than 2 times Pacilio et al.’s study, and T/B was also higher [20]. Compared with the work of Pant et al. [17], we used low-dose CT to correct the SPECT data before measurement, which further restored the true distribution of intrathyroidal radionuclide. Therefore, the 25% threshold automatic delineation method of SPECT/CT can obtain more accurate thyroid volume in GH patients.

There are also some limitations in this study, such as the GH thyroid model volume setting is relatively simple and regular-shaped, which may not fully represent the real GH thyroid and the enrolled cases are limited. In further studies, we will set up a multi-group volume model to simulate GH thyroid and verify the accuracy of the 25% threshold in a larger group of GH patients.

## Conclusions

In conclusion, different T/B had no significant effect on the measurement of thyroid volume in patients with hyperthyroidism; planar imaging method would significantly overestimate thyroid volume in patients with GH. 25% threshold automatic delineation method of quantitative SPECT/CT could obtain more accurate thyroid volume in patients with GH, providing accurate thyroid volume values for individualized doses of ^131^I therapy. While obtaining thyroid volume, quantitative SPECT/CT can also obtain quantitative parameters such as the standardized uptake value (SUV) of ROI in one stop. In the future, various models can be made to combine patient data. To study the quantitative thyroid parameters of SPECT/CT for the quantitative evaluation of different morphology and different functional states of thyroid, we can also try to quantify thyroid cancer ^131^I imaging, evaluate the efficacy, recurrence prediction etc., which has a broad application prospect.

## Data Availability

The datasets used and/or analyzed during the current study are available from the corresponding author on reasonable request.

## Abbreviations

GH: Grave’s hyperthyroidism
T/B: Target-to-background ratios
NEMA: National Electrical Manufacturers Association
IEC: International Electrotechnical Commission
CTAC: CT-based attenuation correction
SC: Scatter correction
RR: Resolution recovery
OSEM: Ordered-subsets expectation maximization
VOI: Volume of interest
SUV: Standardized uptake value
